# Active Volume Control in Smart Phones Based on User Activity and Ambient Noise

**DOI:** 10.3390/s20154117

**Published:** 2020-07-24

**Authors:** V. D. Ambeth Kumar, S. Malathi, Abhishek Kumar, Prakash M, Kalyana C. Veluvolu

**Affiliations:** 1 Computer Science and Engineering, Panimalar Engineering College, Anna University, Chennai 600123, India; vdambethkumar@panimalar.ac.in (V.D.A.K.); smalathi@panimalar.ac.in (S.M.); 2 Department of Computer Science, Banaras Hindu University, Varanasi 221005, India; abhishekryan@bhu.ac.in; 3 Karpagam College of Engineering, Anna University, Coimbatore 641032, India; prakashmohan@kce.ac.in; 4 School of Electronics Engineering, Kyungpook National University, Daegu 41566, Korea

**Keywords:** volume, decibel value, signal, multisensing, social network, sensor introduction

## Abstract

To communicate efficiently with a prospective user, auditory interfaces are employed in mobile communication devices. Diverse sounds in different volumes are used to alert the user in various devices such as mobile phones, modern laptops and domestic appliances. These alert noises behave erroneously in dynamic noise environments, leading to major annoyances to the user. In noisy environments, as sounds can be played quietly, this leads to the improper masked rendering of the necessary information. To overcome these issues, a multi-model sensing technique is developed as a smartphone application to achieve automatic volume control in a smart phone. Based on the ambient environment, the volume is automatically controlled such that it is maintained at an appropriate level for the user. By identifying the average noise level of the ambient environment from dynamic microphone and together with the activity recognition data obtained from the inertial sensors, the automatic volume control is achieved. Experiments are conducted with five different mobile devices at various noise-level environments and different user activity states. Results demonstrate the effectiveness of the proposed application for active volume control in dynamic environments.

## 1. Introduction

In today’s world, smart phones have become integral of part of day-to-day life [1,2,3,4]. Unlike computers, smartphones have applications that run only on a respective Mobile OS’s (like Android, iOS, Windows Fire Flower, etc.) with sensors for various functions [5,6]. However, these devices combines the features of a personal computer and mobile device such as Calling, Texting, etc.; with those of other popular digital mobile devices like personal digital assistants (PDAs), such as media player, video games, GPS navigation, digital camera, digital video camera and event calendar.

In most smart phones, to make the user aware of application functions, several notifications such as ringtones, message tones, app alerts, etc., are employed. These alerts are usually designed as sound notifications to be audible enough for the user to recognize the situation. A device that is placed in a noisy atmosphere requires loud alerts to get the user’s attention. In case the user is in a quiet environment and the device makes the same sound alert, it will be distressing to the user—as well as to the people nearby. Manual intervention by the user is required to adjust the volume by using the volume rocker buttons or by using touch user interface that may not be possible by the user in all situations. Although several sensors are available in the smartphone, automatic volume control based on the ambient environment does not currently exist in most devices.

Several researchers have focused on developing applications for detection of the ambient noise in different environments. In [7], authors developed a system to improve the success of robotic speech presentations for users/listeners in various environments. The system senses a change in its aural relationship with the user and infers the need for an adjustment in its presentation from the user’s listening perspective. The system uses multiple ways to find the correct level of volume by collecting and collaborating all the resources. On the other hand, a new system was developed to investigate the source and context of the noise [8]. By finding the most influential noise in the environment, a grid was created to localize the result. Finally, the system creates a sound map with the help of other noise sources. Further, the system can then extrapolate from the models to the shape of the auditory scene as a whole. Robust recognition of continuous speech by a mobile robot [9] was proposed to perform automatic speech recognition with speakers. A microphone array is utilized with geometric source separation (GSS) along with a post-filter to reduce the interference from other sources. Using this concept, all the sounds in an environment can be easily identified. This approach is specifically designed for multiple source recognition and yet no attempt was made to improve speech recognition of a single source in the presence of environment noise.

There has been a growing interest in the field of human–computer/ human–machine interaction. In [10], authors proposed a system combining various text-to-speech engines to develop a flexible text to speech engine. The new interaction method works exemplarily, but it does not know the context of location or situation it is placed. To overcome this problem, ‘HARK’ robot audition system [11] was developed. The system consists of sound modules, namely source localization, sound partition and automatic speech recognition of separated speech signals that work on any mobile with different microphone configuration. This recognizes the quality of the speaker with the help of speakers mounted on it and can identify the direction and intensity of the sound.

Automatic sound level adjustment has always been a necessity for various reasons. In [12], a system for automatic volume control was developed. In most cases, coworkers in a working environment gets disturbed due to loud sound that is generated when two user communicate accidentally. To overcome this, mobile remote presence (MRP) system [13] was developed to identify the impacts of the sidetone operation in the audio–visual context for annoyance reduction. Automatic volume control for preserving intelligibility [14] was developed to overcome the noise levels of the surroundings that change abruptly or can be transient. Due to repeated occurrences, it is difficult to alter the volume of an audio device by humans to adjust for the changing noise levels. Therefore, a system is necessary to avoid this problem and to analyze the background noise using the microphone in real time. It compensates only for fluctuating noise in the frequency and time domains that interferes with the intelligibility of speech.

Auditory perspective taking [15] is a concept where the system imagines about being in a new environment or place and predicts what they can hear and how it will affect their general comprehension. With this knowledge of another’s auditory perspective, user can adapt to his auditory output to overcome ambient and environmental differences and ensure the quality of the conversation. Robot self-noise (or ego noise), echoes, and reverberation are common interferences that factor in this problem. A prototype robotic interface that uses perspective taking is developed [15] to estimate the effectiveness of its own speech presentation and takes steps to improve intelligibility for human listeners. Here, the robot produces communication more smartly by context awareness and adjusts the volume accordingly. This success confirms the importance of these emerging roadmaps in user interface design, and the developed prototype shows the wide range of skills available to a perspective taking of the robotic interface. Adaptive volume control is one of the important features in mobile application. A new reinforcement learning based application named as RE–IN [16] was developed for android platform for automatic volume control and screen brightness adjustment based on the surrounding noise while playing games. However, the developed application mainly relies on the ambient noise to adjust the volume and do not consider the human activity and state as it was mainly designed for gaming.

Mobile phone user community is required to be aware of sensors function and ability. For this purpose, machine learning has been employed to bridge the gap between the sensor developers [17] and mobile phone users. The knowledge base technique gives a clear picture about the functions of mobile sensors and its durability in various smart phones and provides the user with the required knowledge to select the best sensor for mobile applications. Researchers have shown that mobile sensor sampling algorithm [18] performs best in terms of energy saving as well as mobile area coverage by considering the input under normal walking performed by the human. This algorithm utilizes velocity of human as an important feature for mobile sensing in order to overcome the overlaps caused by spatial regularities. These works highlight the need for development of applications that rely on user activity and user state in order improve the performance of the device and provide comfort to the user.

Various studies show that on average a person spends more time on mobile phones rather than sleeping. A statistical study [19] conducted on hand–phone activities by humans and its popularity among youngsters shows that, the amount of time spent by an average person using mobile phone is very high. If further concludes that the mobile device needs to be smart in order to reduce user interaction as much as possible.

**Figure 1:**
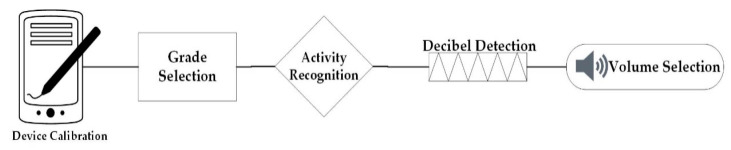
Block diagram for volume control using multisensors.

To overcome the problem of automatic volume adjustment in a smart phone, in this study, an innovative ambient noise activity control method is developed using integrated multi-modal sensing approach. As current generation smartphones usually employs several types of sensors for various applications [20,21,22,23,24], these sensors can be utilized to identify human activity and ambient environment. The proposed system will adjust the volume according to its environment, based on the decibel level that is measured through the microphone. Moreover, the software is developed as an application and will be useful in public places, industrial sites, transportation, hospitals, business meetings, libraries etc. Figure 1 depicts the stream of multisensors employed. The prime objective of this application is to adjust the sound level according to the environment without user intervention. This has the following advantages:

- Eliminates the user need for pressing the volume button;
- Identify a suitable volume level automatically for dynamic environment;
- Makes the mobile device more context-aware for volume adjustment.

In what follows, the proposed methods are discussed in Section 2. Section 3 presents the results. Discussion is provided in Section 4 and Section 5 concludes the study.

## 2. Methods

**Figure 2:**
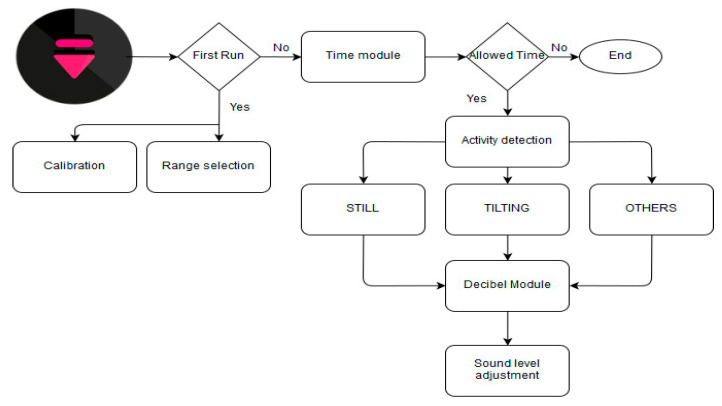
Proposed framework for automatic volume control.

For the development of the proposed system, the Android platform was chosen due to its market share and popularity. Figure 2 presents the proposed framework with multisensors. When the user presses the image button, the program checks for the first-time execution. If so, the range selection and the calibration module will be executed correspondingly. Else, the time module will declare the program to execute in the given time. Thereafter the activity recognition modules recognize the activity of the user and the decibel module will measure the ambient decibel level accordingly. Finally, using these inputs, the sound level selector module adjusts the sound level according to the ambient setting. In what follows, the key steps in Figure 2 are organized as subsections accordingly.

### 2.1. Device-Calibration Algorithm (Algorithm 1)

Every device has its own hardware configuration. To make the program compatible with various devices, calibration must be performed to the device’s microphones. First, a sample value is measured in a calm place (measured in decibels). According to the noise level chart [15], a calm place should have less than 15 dB of sound. A test value is pre–decidedly stored in the program. By comparing the test value with the sample volume, the calibration value is derived. Further, the sound level is measured in decibels (dB).
**Algorithm 1: Device-Calibration**Step 1: get current decibel value Step 2: if decibel value < 15 or decibel value = 15  Calibration value = 0  else if decibel value > 15  Calibration value = decibel value- 15 Step 3: return calibration

**Figure 3:**
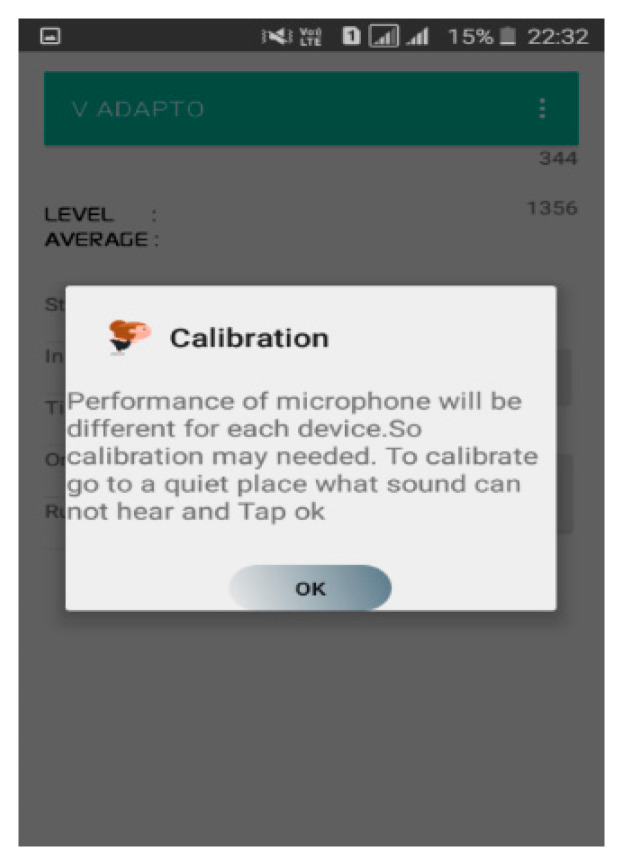
Calibration dialog window.

**Figure 4:**
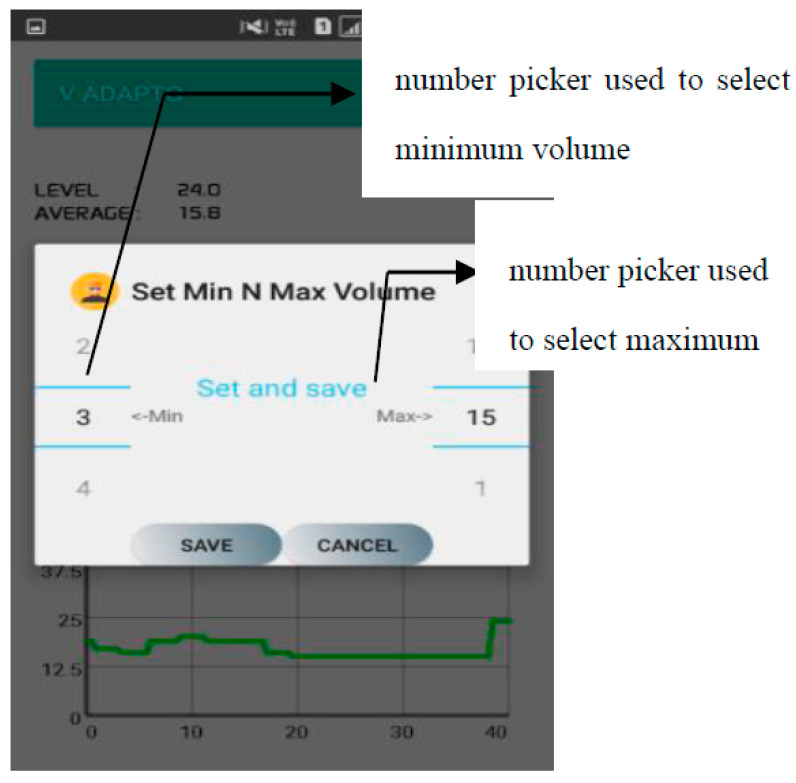
Minimum and maximum selector.

Figure 3 shows the execution of calibration process and Figure 4 depicts the dialog box used to select the minimum and maximum volume. The dialog box contains two number picker and two buttons. The volume level is classified into five levels, namely, very low, low, medium, high and very high.

### 2.2. Grade-Selection Algorithm (Algorithm 2)

During the initial usage, the user needs to specify the range of volume (minimum and maximum values) according to the user’s requirement in the respective number pickers. The first number picker is used to specify the minimum volume whereas the second number picker is used to specify the highest volume level the user can bear. The grade is selected as follows:

*Grade* = (*Max_V* − *Min_V*)/*n* (1)

where *n* is the number of levels, *Min_V* and *Max_V* are the user selected numbers in number picker 1 and picker 2, respectively.

Therefore, the first number picker ranges from 1–15 and this range is already predefined in the mobile application. Moreover, the user has to select a volume level greater than the minimum level so that there will be no mathematical error in the identification of the level.
**Algorithm 2: Grade-Selection****Input:** integer values from number pickers **Output:** the size of the level
Step 1:Open dialogue with number picker 1 and numberPicker 2Step 2:Get the selected number from number picker 1 and storeas minStep 3:get a selected number from number picker 2 and store asmaxStep 4:if min is greater than maxDisplay an error saying “minimum volume can’t behigher than maximum”Step 4.1:elseTotal = maximum – minimumStep 5:level = total/5

In this algorithm, the time unit checks whether the program runs at a specified time given by the user. From the time picker, the starting and the ending time of the program are received. The values from the time picker are in 12 h format. To receive the current time, the system calls the calendar method. This method returns the current time of the device in 24 h format. To match this data, Typecasting is employed to convert different data types into integer (minutes).

The running time for the program is indicated by From_time and To_time. This To _time is derived from the second time picker module since it is in the 12 h format (HH/MM/ (am_pm)). If the selected time is in the form of (HH/MM/pm) the following formula is used.

*T**τ*** = (*Hours* × 60 + *minutes*) + *P* (2)

where *P* is 12*60. Otherwise, if the time specified in (HH/MM/am), then this is used for typecasting.

*T**τ*** = (*Hours* × 60 + *minutes*) (3)

The following equation is used if the current time is typecast to minutes.

*C**τ*** = (*hours* × 60 + *minutes*) (4)

The program will execute if the current time is between From_time and To_time. If the current _time is greater than From _time and lesser than To _time, the program quits itself with a message "time up".

**Figure 5:**
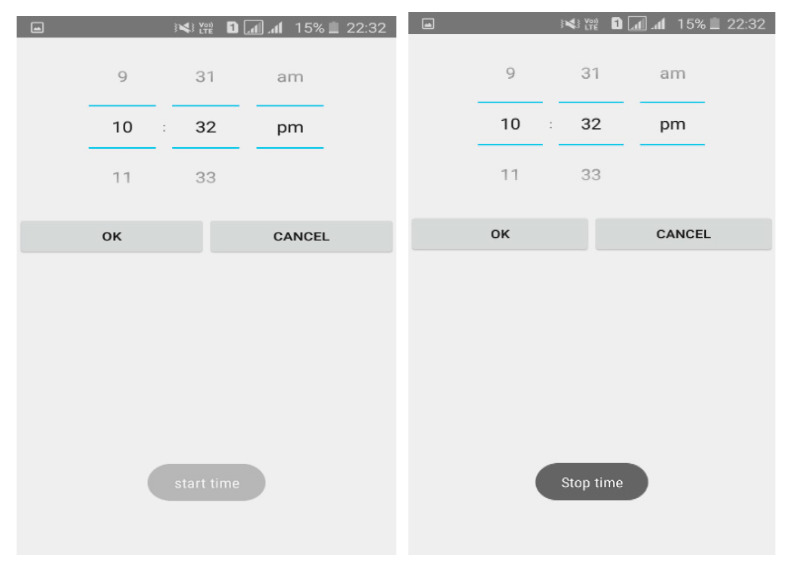
Start time and end time. (**a**) Start time (**b**) Stop time.

The user provides the start time and stop time as inputs in 12 h format as shown in Figure 5. 

### 2.3. Activity-Recognition Algorithm (Algorithm 3)

The activity-recognition algorithm identifies the current activity of the user. The activities can be classified into simple and complex activities. Activities such as walking, walking on stairs, jogging, jumping, driving a motorcycle, driving a car or holding a mobile in hand and placing the mobile on table are known as simple activities. Commonly used sensors in mobile phones are accelerometer [25,26], gyroscope [27], magnetometer [28,29], proximity sensor, light sensor [30], barometer [31,32], thermometer, air humidity sensor [33,34], etc. The user activity can be recognized with the help of these sensors. Accelerometer sensor measures the acceleration in any axis. However, by moving the device in any axis, the sensor data will spike. The device’s orientation along its three-axis can be sensed by the gyroscope sensor [35]. The acceleration can be measured along three spatial axis at any time instant as

𝐴𝑐𝑐𝑖 = < 𝑥𝑖, *yi*, *zi* >, = 1,2,3,…; (5)

The unit of x, y and z are in g-force [36]. In the simple walking activity, if the xi value of Acci spikes for a certain time with short intervals, the device recognizes that the user is moving forward step-by-step. Similarly, if the device is idle there will not be any spikes in any Acci value and, but this occurs under two cases

Case 1: acci = < 0,0,0 > &gyro = < 180,0,0 >

If the device is idle and tilted 1800, it can be inferred that the device is kept on a table.

Case 2: acci = <0,0,0 > &gyro = < (90–180),0,0 >

If the device is idle and tilted 900 to 1800 degree, based on the test set we assume that the user is holding the device in hand. This activity recognition module is designed to identify the following simple activities like STILL—the device is placed anywhere outside; TILTING—user holds the device; and OTHERS—represents any other detected activity. Google activity recognition API is employed for user activity identification.
**Algorithm 3: Activity-Recognition Algorithm****Input**: crisp values from sensors **Output**: Detected activity
Step 1:Get ACTIVITY_RECOGNITION permission from theUser If permission granted go to step 2.Else go to decibel moduleStep 2:Connect with Google Activity Recognition APIStep 3:Run method request Activity UpdatesStep 4:Check if a confident level of detected Activity.STILL = 100%,Put detected_activity = STILLStep 4.1:Else if confident level of detected Activity.TILTING = 100%,Put detected_activity= TILTINGStep 4.2:else if detectedActivity = OTHERSPut detected_activity= OTHERSStep 5:return detected_activity.

### 2.4. Decibel-Detection Algorithm (Algorithm 4)

Using the microphone, a short burst of audio is recorded and saved in the ”*.avi” format. Then the “.avi” file is analyzed to identify the maximum amplitude (hertz per second) that ranges between 20 Hz to 2 kHz in a typical mobile phone. The decibel unit runs continuously to find the current decibel for each second when the decibel module is executed. For each iteration, 500 ms of audio is recorded and analyzed to compute the current decibel value. This decibel value is then sent to the volume selector unit.
**Algorithm 4: Decibel-Detection Algorithm****Input**: short bursts of microphone recording **Output**: Decibel level
Step 1:Create and declare variable decibel and amplitudeStep 2:Create a self-looping thread with 500 ms intervalStep 3:Start media recorder with a microphone as sourceStep 4:For each iteration get amplitude from the mediarecorderStep 5:Decibel = Log_10_ (amplitude/32000)Step 6:Decibel = decibel value—calibration valueStep 7:return decibel

### 2.5. Volume-Selection Algorithm (Algorithm 5)

Sound output in android is classified into these major categories

- Alert sound;
- Notification sound;
- Media volume.

This selector unit adjusts the volume of these sounds according to the crisp values of activity recognition and decibel unit. If the device is in the user’s hand and active; it concludes that the user already has the attention of the device. At this time, the device need not make an alert noise for notification or ringtone. The value TILTING indicates that the device is in the user’s hand and active. In case of detected activity is STILL (the device is idle and resting on a place) then the device needs to make an alert sound to get the user’s attention. Therefore, when the system is in STILL state or another state, the system should make a sound. The volume of this sound must be moderated according to the ambient setting.

The decibel value from the decibel unit is used to decide the volume level of the device. If the decibel value ranges from 10 to 20, it indicates that the device is in a relatively quiet place, the device can get the user’s attention by using minimum volume. The volume escalates as the decibel level increases in the surrounding. If device is placed in a noisy or crowded environment, then the decibel volume will be high and hence the device needs to increase the sound level in order to get user’s attention. This unit will adjust the notification, ringtone and media volume at the same time if the device is in STILL or OTHER state. However, in TILTING state, only the media volume is adjusted.

The volume selector is executed every 10–20 s based on the activity. If the activity is identified as STILL, the volume selector gets the average decibel for 20 s and then it changes the volume level accordingly. For any other activity other than TILTING, the volume selector is executed for every 10 s. The volume selection is identified by the following equation:

(6) $$V_{si}=\overset{n}{\underset{i=1}{\sum }}min+G*i$$

where

(7) $$V_{s0}=min$$

and *G* is the level and ‘i’ is the position.
**Algorithm 5: Volume-Selection Algorithm****Input**: detected activity, the decibel level **Output**: volume level selection If detected activity is TILTING**{** set(ringtone, Notification) = 0; for each I = 0 to 20 ( sum<− sum+decibel ) Average<−sum/20 Switch (average)   Case 0–10: media volume < −$V_{s0}$   Case 11–20: media volume< − $V_{s1}$   Case 21–30: media volume < − $V_{s2}$   Case 31–40: media volume < −$V_{s3}$   Case >40: media volume <− $V_{s4}$} Else {if detected activity is STILL( put N < −20 ) Else  (put N < −10) for each I = 0 to N sum = sum + decibel Average = sum/N end Switch (average) {   Case 0–10: All volume< − $V_{s0}$   Case 11–20: All volume< − $V_{s1}$   Case 21–30: All volume < − $V_{s2}$   Case 31–40: All volume < − $V_{s3}$   Case > 40: All volume < − $V_{s4}$ }}

## 3. Results and Discussion

For experimental evaluation, five mobile phones from different makers—Samsung galaxy j2, Moto, HTC, Sony and Blackberry—were employed. The developed algorithms were embedded within the software in the form of a mobile application. The software relied on the inbuilt hardware available in the mobile phones for activity detection and volume detection. The application was tested in various environments and in different user activity states. The calibration was performed in a calm environment at various locations such as the living room, residential area and library.

**Figure 6:**
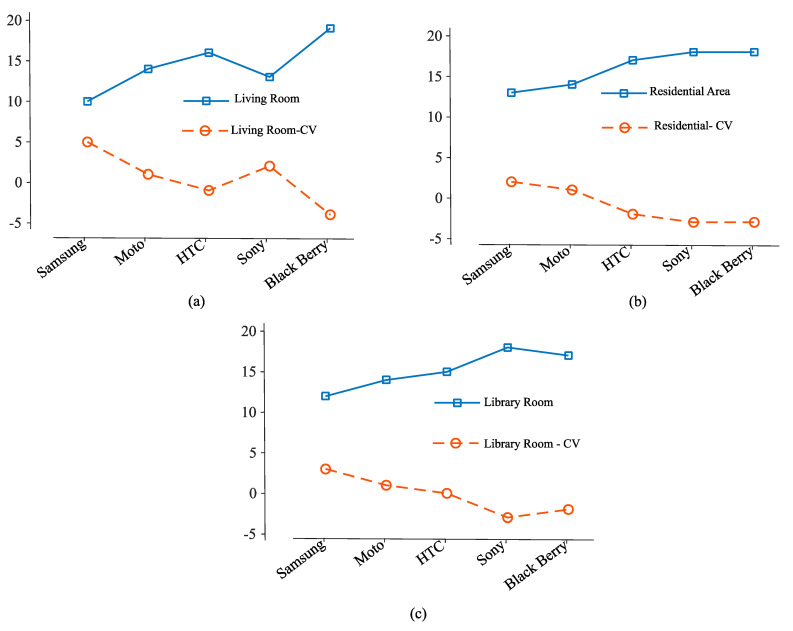
Decibel value of calibration process for (**a**) Living room; (**b**) residential area; (**c**) library.

Figure 6. depicts the outcome of the calibration process in three different environments where noise levels are different. The calibrated value shows small variations in the living room and library due to low noise level across different devices. The volume level ranges from 0 to 15 and the user has to choose between two values—minimum and maximum. In total there can be 120 combinations. The first one must be less than others, and these values are used to find the value of grade as follows:

Grade = round of ((max − min)/4 )(8)

The proposed system considers sound in five different levels, namely very low, low, medium, high and very high. As sound level varies for different values of user inputs, appropriate grade is assigned.

**Figure 7:**
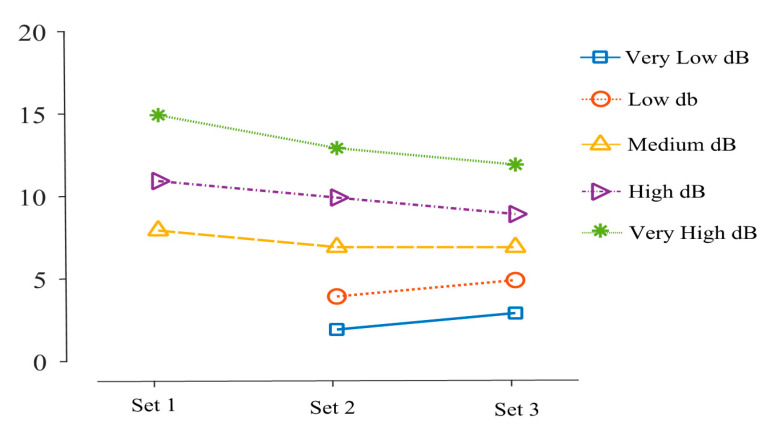
Volume level for selected sets.

Figure 7 shows the volume level value for three sets of min and max values like Set 1 = (1, 15), Set 2 = (2, 13) and Set 3 = (3, 12). The volume selector unit selects the volume level according to the decibel level and detected activity. It is executed for every 10 s or 10-decibel averages according to the detected activity. To evaluate the program, various time averages are considered for analysis by considering different activities. For tilting and still activities, the decibel level of 20 is chosen. For every 10 s, the volume selector is executed.

**Figure 8:**
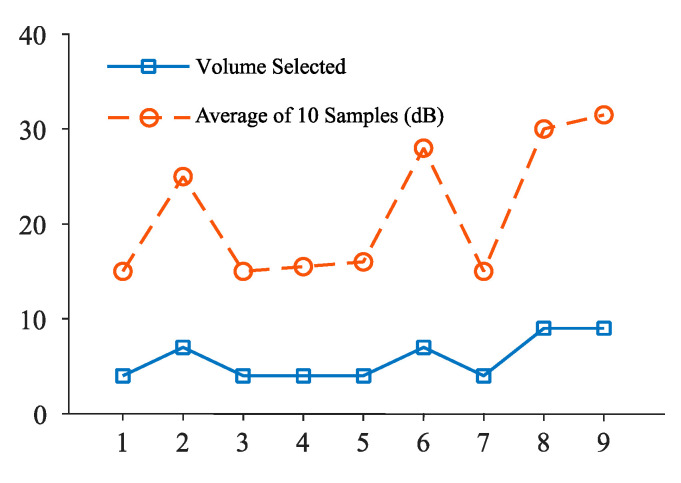
Volume selection for 10-decibel-level inputs.

**Figure 9:**
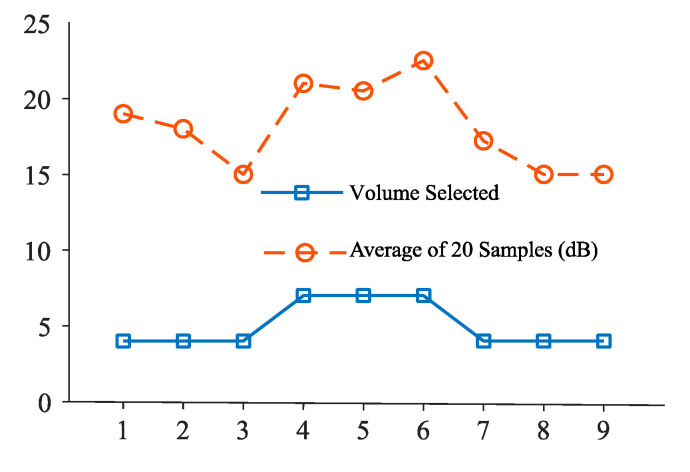
Volume selection for 20-decibel-level inputs.

**Figure 10:**
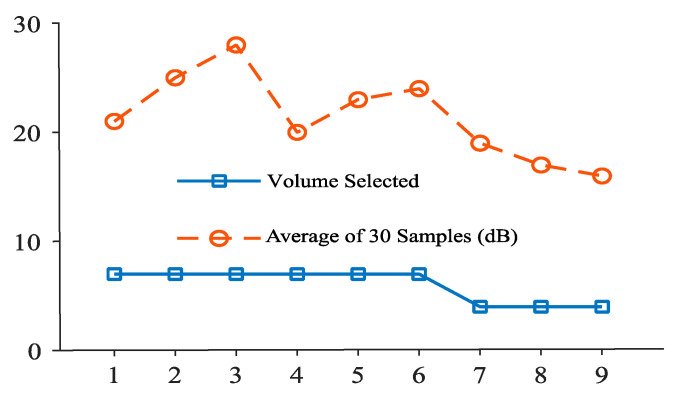
Volume selection for 30-decibel-level inputs.

Figure 8 represents the decibel selector that is executed for every 10 samples in an open park environment. From this, it is clear that the ambient volume level changes frequently and causes annoyance to the user. Figure 9 depicts the volume level selection for 20 samples average. Although the volume change is minimum, there is a higher chance of user listening to the media at an unsuitable volume level. Figure 10 shows the volume level selection for 30 samples average. The frequency of volume changed is low and the chance of user listening to media at correct volume is high. Based on the analysis, 20 samples are chosen to be more appropriate for the tilting activity. For other activities, the number of samples chosen is 10 since the activity is unknown. To overcome this problem, the volume selector is executed every 5 s.

**Figure 11:**
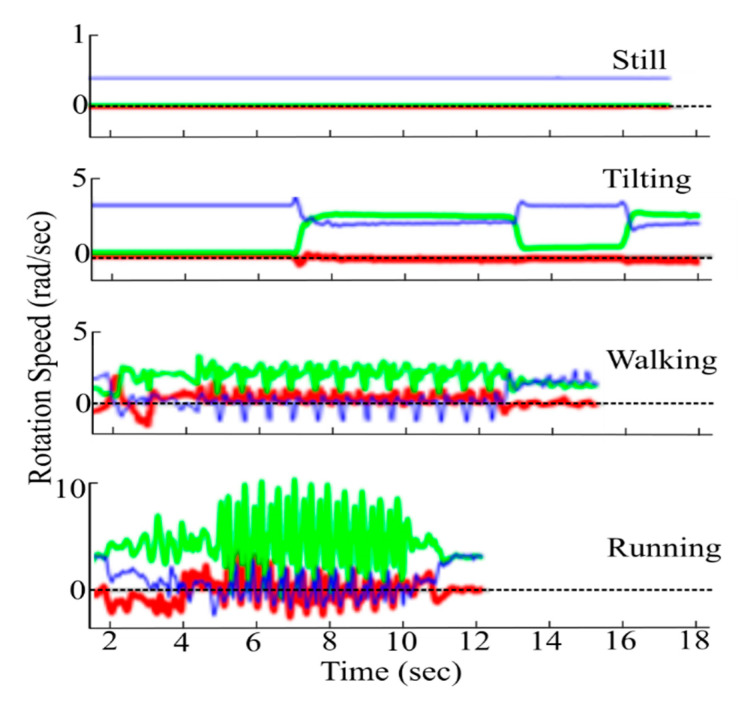
Test case three-axis outputs for various activities.

Tests are also conducted to verify the activity recognition based on the activity detection API where the input is obtained from the gyro sensor in the device. Four activities (still, tilting, walking and running) are performed for validation to verify if the software detects the correct activities. Figure 11 shows the sensors outputs in three axes x, y and z-axis for different activities. The activity detection API relies on the variations in the sensor outputs to identify these activities as shown in Table 1 Although several activity states can be included in the algorithm, for illustration only four activities are chosen. More number of activities may also be included for better understanding about the activity recognition such as climbing, falling, running, etc. In some cases, complex activities like driving can be also considered to test the algorithms. When the detected activity is TILTING, the ringtone and notification is set as zero and only the media sound level is changed.

**Figure 12:**
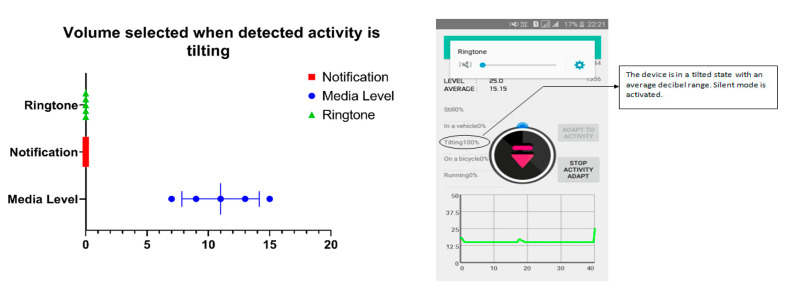
Volume selection during tilting.

Figure 12 shows the tilting state activity where the ringtone and the notification volume are set as zero. According to the algorithm, all sound levels should be selected minimum. When the detected activity is still or other; volume is changed according to the surrounding volume level.

**Figure 13:**
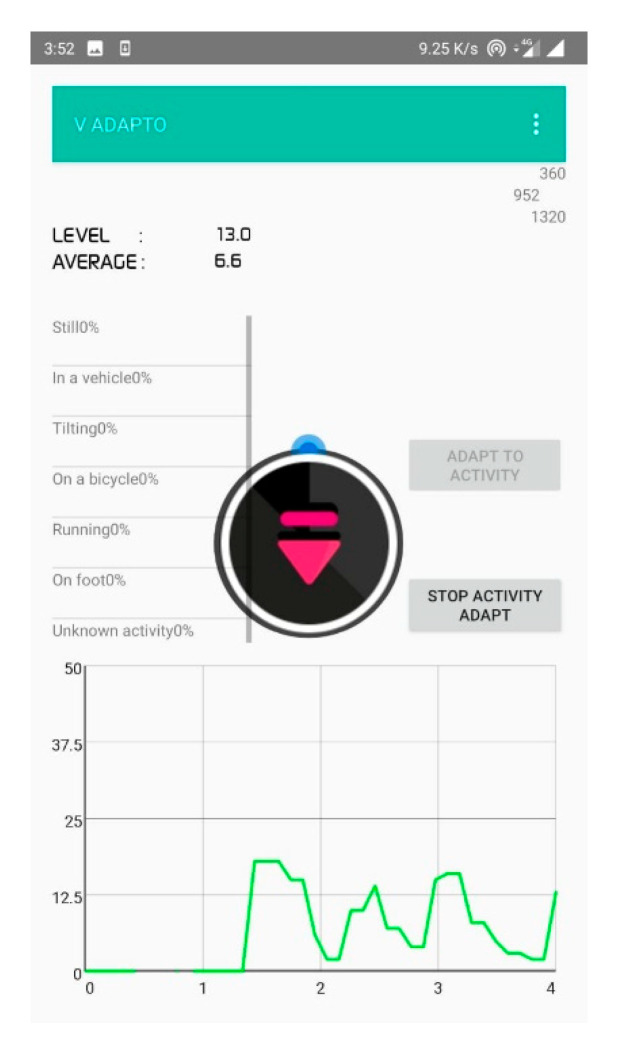
Average Level below 10.

**Figure 14:**
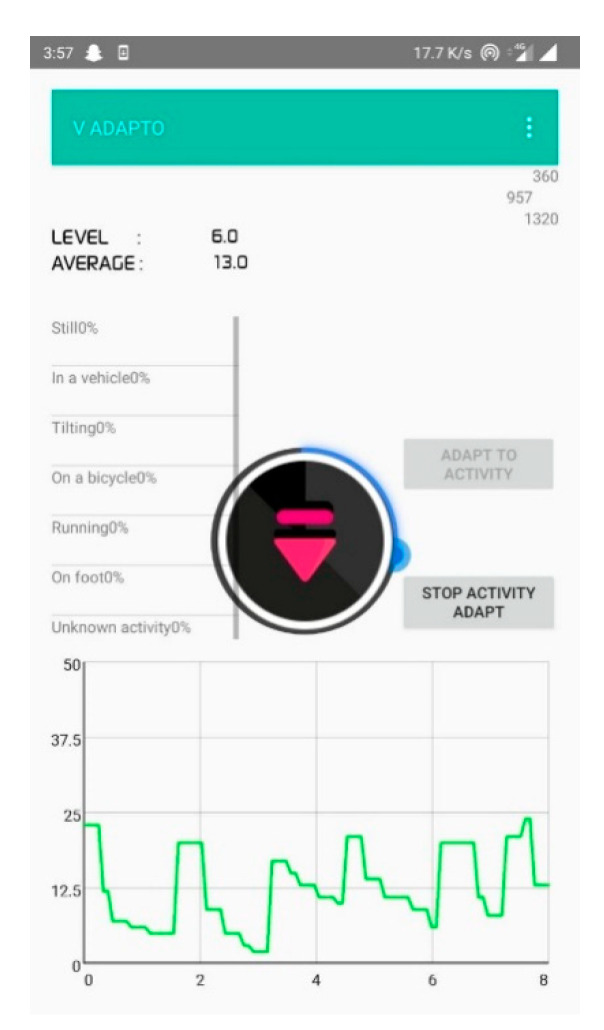
Average level below 20.

**Figure 15:**
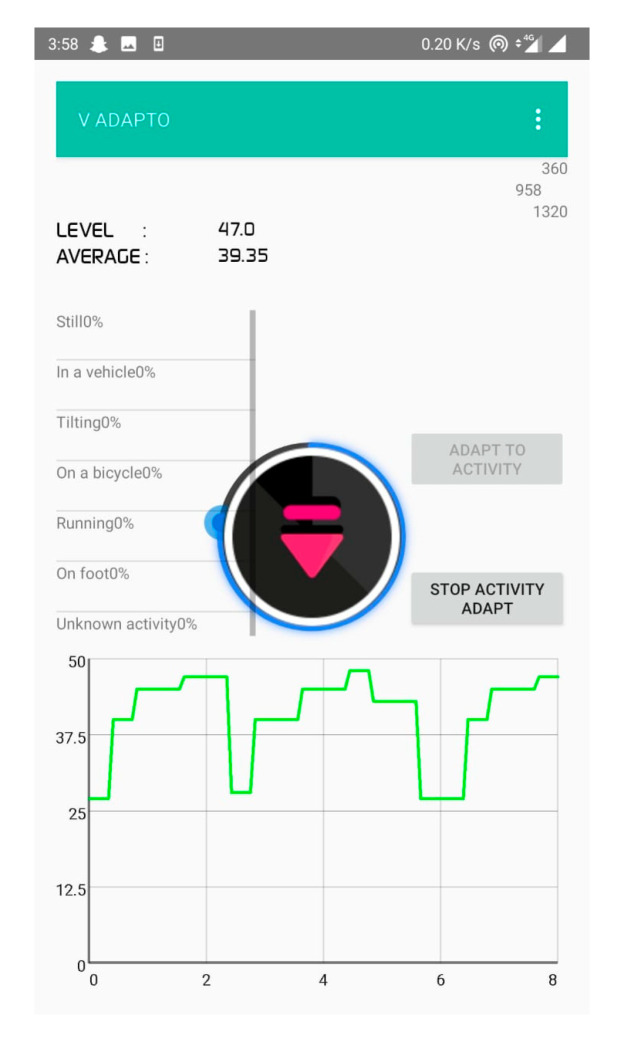
Average level below 40.

Figure 13, Figure 14 and Figure 15 represents various average decibels levels. When the average sound level is considered low and the detected activity is still, the volume level selector algorithm increases the sound level by one grade. In case, if the surrounding sound level average is above 25, the sound level selector algorithm selects the higher level. As the ambient sound level increases, the device sound level is increased in order to make the device sound audible for the user. The Maximum sound level is selected when the decibel level of the surrounding environment goes above 50. A Decibel level above 50 represents that the ambient sound level is noisy for the user. Therefore, the device selects the highest possible volume level to grab the user attention in the case of notification or ringtone. Taking into account the different manufactures of the mobile phones together with their characteristics, the decibel value for a user may differ. Hence user feedback and selections of minimum and maximum volume levels plays in key role in the identification of a comfortable volume level. The comparison of the proposed system with existing systems is tabulated in Table 1.

From the comparative analysis, it is clear that the proposed system offers various features when compared to the existing methods. Moreover, the developed algorithm provides greater accuracy to detect the activity and sound level in real time.

## 4. Discussion

In this study, different algorithms are developed in the proposed system—Grade-selection algorithm for computing execution time; Activity-recognition algorithm that fuses various sensors for identification of user activity; Decibel-selection algorithm for effective sound level measurement in both device and surrounding environment; Volume selection for adjusting the volume automatically based on the ambient noise level. This proposed application was tested at various locations with five different sound levels. Based on the tests conducted with several mobile devices, the sound level in the device was regulated to be within the comfort level of the users. Moreover, the system performed well for low noise level and high noise levels as shown in Figure 8. When the application identifies the unknown user activity, the time interval is reduced to five seconds. For noise levels greater than 15 decibels, the volume selector module automatically regulates the volume to the user comfortable level.

As compared to the manual systems [36,38] where the user intervention is required, this system performs better even in commercial areas with higher noise levels. Most of the existing methods [13,16] focus on a specific application and are not applicable to various environments and user activities. In comparison, the proposed work relies on user activity and ambient noise levels from various environments for automatic volume control. More types of activity can be included to further improve the application scope and performance that adapts more to the user lifestyle.

Different people may have different motion intensities and individual comfort levels. In the present scenario, the application only relies on the type of the activity and not the intensity level. The effect of user intensity level on the volume is yet to be analyzed and will be a topic for our future research.

The proposed system also does not rely on GPS sensor data and google map location to identify the environment. Instead, with the help of sensors it identifies the noise level and identifies the activity and thereby automatically adjusts the sound level accordingly according to its dynamic state. Future work will rely on including GPS location data to further improve the application scope and performance.

## 5. Conclusions

An automatic volume control application for a smartphone was developed in this study. The approach relies on built-in sensors to estimate the user state and the ambient noise level to automatically adjust the volume of the device. Further, the device also allows for user customization as it relies on the user calibrated inputs to adjust the volume. The proposed system is evaluated in various locations under different noise levels. Results show that application works effectively in different indoor and external environments under various decibel levels. In comparison with existing methods, the proposed method not only relies on the ambient noise, but also relies on the user activity state to decide on the volume level. The proposed system can work efficiently as an inbuilt function as a part of AI-based services in mobile devices such as “Siri’’ or any Android-based application. Furthermore, this development can also eliminate the need for volume button in the future mobile devices and this may provide more space to other IC components leading to the revolution in the smartphone design.

## Figures and Tables

**Table 1:** Comparison of the proposed system with existing systems.

Application	Automatic Volume Change	Calibrate Function	Sound Level Change Based on Output	Set Profile Based on Activity	Dynamic Volume Change
Volume control [36]	No	No	Yes, not automatic	No	Yes
Smart volume control [37]	Yes	Yes	Yes	No	No
Sound assistant [38]	No	No	Yes, not automatic	No	Yes
Eric Qing Li [39]	No	No	No	Yes	No
Alex Boudreau [40]	Yes	Yes	No	No	No
Kenneth Louis Herman [41]	No	No	No	No	No
Proposed method	Yes	Yes	Yes	Yes	Yes

## References

[1] http://www.dailymail.co.uk/health/article-2989952/How-technology-taking-lives-spend-time-phones-laptops-SLEEPING.html.

[2] Allouch A., Koubâa A., Abbes T., Ammar A. (2017). Road Sense: Smartphone Application to Estimate Road Conditions Using Accelerometer and Gyroscope. IEEE Sens. J..

[3] Chen Y., Shen C. (2017). Performance Analysis of Smartphone-Sensor Behavior for Human Activity Recognition. IEEE Access.

[4] Xing S., Tong H., Ping J. (2014). Activity Recognition with Smartphone Sensors. Tsinghua Sci. Technol..

[5] Garcia-Ceja E., Osmani V., Mayora M. (2016). Automatic Stress Detection in Working Environments from Smartphones’ Accelerometer Data: A First Step. IEEE J. Biomed. Health Inform..

[6] Johan W., Malekian R. (2017). Physical Activity Recognition from Smartphone Accelerometer Data for User Context Awareness Sensing. IEEE Trans. Syst. Man Cybern. Syst..

[7] Brock D., Martinson E. (2006). Robotic speech presentations for individual human user/listeners in relevant circumstances. Aurally Informed Performance: Integrating Machine Listening and Auditory Presentation in Robotic Systems.

[8] Martinson E., Schultz A. Robotic discovery of the auditory scene. Proceedings of the IEEE International Conference on Robotics and Automation.

[9] Valin J., Yamamoto S., Rouat J., Michaud F., Nakadai K., Okuno H.G. (2007). Robust Recognition of Simultaneous Speech by a Mobile Robot. IEEE Trans. Robot..

[10] Michael A.G., Alan C.S. (2008). Human robot Interaction–A survey. Found. Trends Hum. Comput. Interact..

[11] Nakadai K., Takahashi T., Okuno H.G., Nakajima H., Hasegawa Y., Tsujino H. (2010). Design and Implementation of Robot Audition System “HARK”—Open Source Software for Listening to Three Simultaneous Speakers. Adv. Robot..

[12] Fleber F.S. (2015). Voice Detection for Automatic Volume Controls and Voice Sensors. U.S. Patent.

[13] Paepcke A., Soto B., Takayama L., Koenig F., Gassend B. Yelling in the hall: Using sidetone to address a problem with mobile remote presence systems. Proceedings of the 24th Annual ACM Symposium on User Interface Software and Technology.

[14] Felber F. An automatic volume control for preserving intelligibility. Proceedings of the 34th IEEE Sarnoff Symposium.

[15] Martinson E., Brock D. (2013). Auditory Perspective Taking. IEEE Trans. Cybern..

[16] Abeywardhane J.S.D.M.D.S., de Silva E.M.W.N., Gallanga I.G.A.G.S., Rathnayake L.N., Jagath W. Optimization of Volume & Brightness of Android Smartphone through Clustering & Reinforcement Learning. Proceedings of the IEEE International Conference on Information and Automation for Sustainability.

[17] Khokhlov I., Reznik L., Ajmera S. (2020). Sensors in Mobile Devices Knowledge Base. IEEE Sens. Lett..

[18] Thejaswini M., Rajalakshmi P., Desai U.B. (2015). Novel sampling algorithm for human Mobility Based Mobile Phone Sensing. IEEE Internet Things J..

[19] Roberts J.A., Yaya L.H., Manolis C. (2014). The invisible addiction: Cell-phone activities and addiction among male and female college students. J. Behav. Addict..

[20] Jain A., Kanhangad V. (2018). Human Activity Classification in Smartphones using Accelerometer and Gyroscope Sensors. IEEE Sens. J..

[21] Jin R., Shi L., Zeng K., Pande A., Mohapatra P. (2016). MagPairing: Pairing Smartphones in Close Proximity using Magnetometers. IEEE Trans. Inf. Forensics Secur..

[22] Won J.Y., Ryu H., Delbruck T., Lee J.H., Hu J. (2015). Proximity Sensing Based on a Dynamic Vision Sensor for Mobile Devices. IEEE Trans. Ind. Electron..

[23] Jin R., Shi L., Zeng K., Pande A., Mohapatra P. MagPairing: Exploiting magnetometers for pairing smartphones in close proximity. Proceedings of the IEEE Conference on Communications and Network Security.

[24] Cheng H.C., Liu K.C., Hsieh C.Y., Chan C.T. Smartphone-based Pedestrian localization algorithm using inertial and light sensors. Proceedings of the IEEE International Conference on Applied System Innovation (ICASI).

[25] Noise Level Chart. http://www.noisehelp.com/noise-level-chart.html.

[26] Hua J., Shen Z., Zhong S. (2017). We Can Track You if You Take the Metro: Tracking Metro Riders Using Accelerometers on Smartphones. IEEE Trans. Inf. Forensics Secur..

[27] Bisio I., Delfino A., Lavagetto F., Sciarrone A. (2017). Enabling IoT for In-Home Rehabilitation: Accelerometer Signals Classification Methods for Activity and Movement Recognition. IEEE Internet Things J..

[28] Lahdenoja O., Hurnanen T., Iftikhar Z., Nieminen S., Knuutila T., Saraste A., Kiviniemi T., Vasankari T., Airaksinen J., Mikko P. (2018). Atrial Fibrillation Detection via Accelerometer and Gyroscope of a Smartphone. IEEE J. Biomed. Health Inform..

[29] Wahdan A., Georgy J., Noureldin A. (2015). Three-Dimensional Magnetometer Calibration with Small Space Coverage for Pedestrians. IEEE Sens. J..

[30] Andriyan B.S., Danudirdjo D., Antonius D.S., Rahmawati D. (2017). Magnetic Subsurface Imaging Systems in a Smartphone Based on the Built-In Magnetometer. IEEE Trans. Magn..

[31] Leikanger T., Schuss J., Juha H. Calibration of smartphone light sensors with a near field communication enabled reference. Proceedings of the 2016 IEEE Sensors.

[32] Jeon J., Kong Y., Nam Y., Yim K. An Indoor Positioning System Using Bluetooth RSSI with an Accelerometer and a Barometer on a Smartphone. Proceedings of the 10th IEEE International Conference on Broadband and Wireless Computing, Communication and Applications (BWCCA).

[33] Won M., Zhang S., Chekuri A., Son S.H. Enabling energy-efficient driving route detection using built-in smartphone barometersensor. Proceedings of the 19th IEEE International Conference on Intelligent Transportation Systems (ITSC).

[34] Jafari H., Li X., Qian L., Chen Y. Community based sensing: A test bed for environment air quality monitoring using smartphone paired sensors. Proceedings of the 36th IEEE Sarnoff Symposium.

[35] Smith J.P., Li X. AirSniffer: A smartphone-based sensor system for body area climate and air quality monitoring. Proceedings of the 10th International Symposium on Medical Information and Communication Technology (ISMICT).

[36] Netroken Volume Control (Version 4.99.3). https://play.google.com/store/apps/details?id=netroken.android.persistfree&hl=en_IN.

[37] Nexoft Mobile Smart Volume Control-Auto Volume (Version 1.9). https://play.google.com/store/apps/details?id=com.ceyhun.smartvolume&hl=en_IN.

[38] Samsung Electronics, Co. Ltd. Sound Assistant (Version 3.3.08.0). https://play.google.com/store/apps/details?id=com.samsung.android.soundassistant&hl=en_IN.

[39] Li E.Q. Automatic Volume Control Based on Context and Location. https://patentimages.storage.googleapis.com/3b/56/7a/78f5a013c0e83c/US20150011195A1.pdf.

[40] André L., Boudreau A., Gariepy F. Sound Volume Automatic. https://patentimages.storage.googleapis.com/62/36/72/da1a9be8366237/US8116461.pdf.

[41] Kenneth L.H., Dixon M., James B. Dynamic Volume Adjustment. https://patentimages.storage.googleapis.com/71/8a/76/bfa4c7c0e5f978/US6968063.pdf.

